# Retroperitoneal lymph‐node dissection for isolated nodal recurrence of renal cell carcinoma after nephrectomy: Contemporary outcomes of a case series

**DOI:** 10.1002/bco2.70190

**Published:** 2026-06-29

**Authors:** Alberto Costa Silva, Noel Clarke, Aziz Gulamhusein

**Affiliations:** ^1^ The Christie NHS Foundation Trust Manchester UK; ^2^ Faculty of Medicine of University of Porto, RISE—Health Research Network University Hospital Centre of São João Porto Portugal

**Keywords:** lymph node recurrence, renal cell carcinoma, retroperitoneal lymph‐node dissection

## Abstract

**Background and objective:**

Isolated retroperitoneal lymph‐node (RPLN) recurrence after radical nephrectomy (RN) for renal cell carcinoma (RCC) is uncommon, and optimal management remains undefined. This study reports contemporary outcomes of retroperitoneal lymph‐node dissection (RPLND) for isolated nodal recurrence, including in patients previously treated with stereotactic ablative body radiotherapy (SABR) or systemic therapy.

**Methods:**

A retrospective review was performed of all patients undergoing RPLND for isolated nodal recurrence of RCC between July 2023 and October 2024. Clinical, operative, and pathologic variables were collected, and postoperative complications were graded using the Clavien–Dindo system. Recurrence‐free survival (RFS) was calculated from RPLND to recurrence or last follow‐up.

**Results:**

Eight patients met inclusion criteria. Median age at RPLND was 59.5 years, and six were male. The median interval from RN to RPLND was 29 months. Median operative time was 255 min, estimated blood loss 300 mL, and hospital stay 6.5 days. One patient experienced postoperative complication (Clavien–Dindo IIIa). All specimens confirmed malignancy with negative margins. After a median follow‐up of 18 months, three patients (37.5%) recurred, with a mean RFS of 15.3 months.

**Conclusions:**

RPLND is a safe and viable option for isolated nodal recurrence of RCC, offering complete resection and oncologic outcomes comparable to historical data, and may serve as an important component of multimodal management.

## 1 INTRODUCTION

Renal cell carcinoma (RCC) accounts for approximately 3% of all adult malignancies. When detected at a localized stage, surgical management achieves 5‐year cancer‐specific survival rates around 90%.^1^ Despite curative‐intent radical nephrectomy (RN), disease recurrence occurs most often at distant metastatic sites but a subset of patients, estimated at 1%–3%, experience isolated retroperitoneal lymph‐node (RPLN) recurrence.^2^ The optimal management of RPLN recurrence remains undefined. In current practice, treatment strategies typically involve systemic therapy or stereotactic ablative body radiotherapy (SABR), either alone or in combination, although supporting evidence remains limited.^3^, ^4^, ^5^

In this setting, the role of retroperitoneal lymph‐node dissection (RPLND) in patients with isolated nodal recurrence remains insufficiently defined, mainly due to the scarce number of available studies, and earlier series were conducted before the widespread use of SABR and modern systemic therapies, namely immunotherapy.^6^, ^7^, ^8^, ^9^

We aimed to evaluate perioperative and oncologic outcomes of RPLND for isolated nodal recurrence after RN in the modern therapeutic era.

## 2 METHODS

Ethical approval was waived by the local ethics committee in view of the retrospective nature of the study and all the procedures being performed were part of the routine care. Informed consent was obtained from all individual participants included in the study.

All consecutive patients who underwent RPLND for isolated lymph‐node recurrence of RCC after RN between July 2023 and October 2024 were included. Clinical information was retrospectively obtained from institutional medical records. Recurrences were identified on follow‐up chest/abdomen/pelvis computed tomography (CT). Patients with distant metastases were excluded.

**FIGURE 1:**
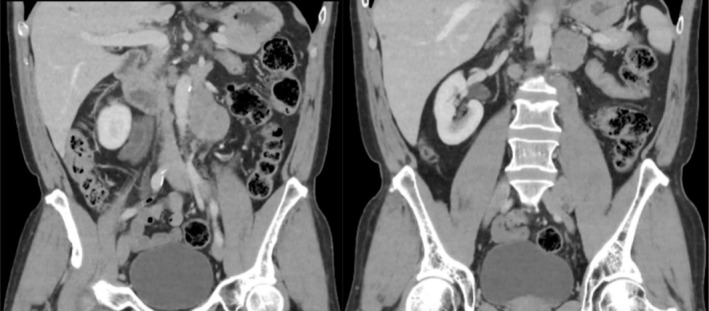
Retroperitoneal lymph node recurrence involving para‐aortic nodes and the renal hilum following left radical nephrectomy.

All cases were reviewed in a dedicated renal multidisciplinary team meeting before proposal to RPLND. There were no predefined selection criteria for proceeding with RPLND; decisions were made on a case‐by‐case basis, considering patient performance status, disease resectability, and patient preference. RPLND was also offered to selected patients with disease progression following prior SABR and/or systemic therapy. RPLND was performed using modified surgical templates, tailored to the site of recurrence as right‐sided, left‐sided, or bilateral.^10^ All cases were performed using an open approach due to prior surgery, radiation, and disease volume (Figure 1).

Collected data included sex, age, and body mass index at time of RPLND, tumour laterality, histopathology report in RN specimen, interval from RN to first node recurrence, and to RPLND. For RPLND, the following variables were analysed: surgical template used, operative time, estimated blood loss (EBL), length of hospital stay (LOS), and major postoperative complications, classified according to the Clavien–Dindo system (≥ grade III). Post‐RPLND outcomes included recurrence, recurrence‐free survival, and the type of subsequent treatment.

Data were collected and analysed with the Statistical Package for the Social Sciences (version 30; IBM, USA). The Shapiro–Wilk test was used to assess the normality of the parameter distribution. Normally distributed continuous variables are expressed as the mean ± standard deviation, and nonnormally distributed variables are expressed as the median (25th–75th percentile).

## 3 RESULTS

Eight patients were identified. Individual patient data are presented in Table 1. The median age at RPLND was 59.5 (28.0–33.8) years and six patients (75%) were male. The primary tumour was right sided in six cases (75%). RN histopathology report stage was pT3a in five patients (62.5%), pT2b in one (12.5%), and pT1b in one (12.5%). Two patients (25%) were pN1, one (12.5%) pN0, and five (62.5%) pNx. Clear‐cell carcinoma was the most commons subtype in three patients (37.5%), with four patients (50%) having high‐grade disease. The median interval time from RN to first node recurrence and from RN to RPLND was 11.5 (8.3–25.0) and 29.0 (18.3–45.5) months, respectively. The nodal recurrence size before RPLND was 42.5 (32.5–57.8). Four patients (50%) had previously received SABR or systemic therapy. Unilateral templates were used in six (75%) patients.

**TABLE 1:** Individual characteristics of patients undergoing retroperitoneal lymph‐node dissection (RPLND) for isolated nodal recurrence of renal cell carcinoma after nephrectomy.

Age*, BMI*, Gender	RN histology	Interval RN to first recurrence, *months*	Interval RN to RPLND, *months*	Previous SABR or systemic therapy	Recurrence location and size*, mm*	Surgical time*, hours*	RPLND Template	EBL*, mL*	LOS, days	Complications	RPLND histology	Post‐RPLND relapse and treatment
39 years old, 38 kg/m ^2^ Male	Left pT3aN1, Fumarate hydratase deficient Sarc/Rhab−	7	48	RN ➔ RPLN recurrence 7 months after ➔ Ipi + Nivo ➔ SAB R ➔ RPLND	Para‐aortic; 60 × 50	240	Left	100	9	0	=	0
57 years old, 28 Male	Left pT3aN0, Chromophobe Sarc/Rhab− Leibovich 6	10	11	0	Para‐aortic, interaortocaval, retrocaval; 45 × 32	360	Bilateral	300	5	0	=	0
71 years old, 24 kg/m ^2^ Male	Right, not known **	428	430	0	Paracaval, retrocaval; 40 × 24	210	Right	100	6	0	Adenocarcinoma	0
52 years old, 40 kg/m ^2^ Female	Right pT1bNx, Papillary Sarc/Rhab−; ISUP 3 Leibovich 2	13	38	RPLN recurrence 13 months after RN ➔ Cabo ➔ SABR ➔ RPLND	Interaortocaval, retrocaval; 48 × 24	360	Right	300	7	0	=	0
59 years old, 34 kg/m ^2^ Male	Right pT3aNx, Clear cell Sarc/Rhab−; ISUP 4 Leibovich 8	9	29	RN ➔ Adjuvant Pembro ➔ RPLN recurrence 9 months after RN ➔ Cabo ➔ side effects ➔ RPLND	Paracaval; 61 × 50	240	Right	100	5	0	=	0
65 years old, 32 kg/m ^2^ Male	Right pT3aNx, Clear cell Sarc/Rhab−; ISUP 4 Leibovich 7	28	29	0	Paracaval; 30 × 24	360	Right	500	15	Fever (CD II)	=	Progression to mediastinum 10 months after RPLND
64 years old, 33 kg/m ^2^ Male	Right pT2bNx, Clear cell Sarc/Rhab+; ISUP 4 Leibovich 8	16	19	0	Para‐aortic, interaortocaval, retrocaval; 55 × 20	180	Bilateral	800	13	Chyle leak with drain insertion (CD IIIa)	=	Relapse 11 months after RPLND ➔ Axi + Av
60 years old, 28 kg/m ^2^ Female	Right pT3aN1,Chromophobe Sarc/Rhab− Leibovich 1	8	18	RPLN recurrence 8 months after RN ➔ SABR ➔ RPLND	Interaortocaval, paracaval; 40 × 24	270	Right	500	5	0	=	Relapse 24 months after ➔ second RPLND

* at RPLND.

** long gap between events, no records from nephrectomy report.

Abbreviations: −, absent/negative; +, present/positive; same histologic result as nephrectomy. Av, avelumab; Axi, axitinib; BMI, body mass index; Cabo, cabozantinib; CD, Clavien–Dindo classification; EBL, estimated blood loss; h, hours; Ipi, ipilimumab; ISUP, International Society of Urological Pathology grade; Leibovich, Leibovich risk score; LOS, length of hospital stay; mL, millilitres; mm, millimetres; Nivo, nivolumab; Pembro, pembrolizumab; pT/pN, pathologic tumour/nodal stage; RCC, renal cell carcinoma; Rhab, rhabdoid differentiation; RN, radical nephrectomy; RPLND, retroperitoneal lymph‐node dissection; SABR, stereotactic ablative body radiotherapy; SABR, stereotactic body radiotherapy; Sarc, sarcomatoid differentiation.

The median operative time was 255 (218–360), the median estimated blood loss was 300 (100–500), and the median hospital stay was 6.5 (5.0–12.0) days. One patient (12.3%) experienced postoperative complications: chyle leak requiring drainage (Clavien–Dindo IIIa). All pathologic findings at RPLND specimens were positive for malignancy with negative margins.

During a median follow‐up of 18 months (14–22 months) after RPLND, three patients (37.5%) developed further recurrence—two distant and one locoregional—with a mean recurrence‐free survival of 15.3 ± 7.6 months from RPLND.

## 4 DISCUSSION

RPLND in RCC may serve a role both at the time of RN and in the management of nodal recurrence. In the first scenario, evidence has not been shown to provide an overall survival benefit.^11^, ^12^ Evidence for RPLND in isolated nodal recurrence remains scarce. The paucity of existing data on this cohort probably reflects both the relative infrequency as well as clinicians' historical reluctance to consider resection in these cases.

Perioperative outcomes in this series aligned with previously published data. The median operative time was 255 min, estimated blood loss 300 mL, and hospital stay 6.5 days—values consistent with reported ranges of 225–270 min, 300–575 mL, and 5–6 days, respectively.^6^, ^7^, ^8^, ^9^ The overall complication rate was 12.3%, with only one Clavien Dindo ≥ III and no operative mortality, comparable to prior series reporting 27%–37.5% overall and 5%–10% major complications.^6^, ^7^, ^8^, ^9^ These findings support that RPLND remains a safe procedure when performed in selected patients within experienced centers.

Our results align with previously published series, showing a median interval from radical nephrectomy to RPLND of 29 months (vs. 10–26 months in prior reports) and a mean recurrence‐free survival of 15.3 months (vs. 9.6–19.5 months using similar definitions). After RPLND, 37.5% of patients developed disease recurrence, consistent with the 30%–60% rates reported.^6^, ^7^, ^8^, ^9^

All pathology findings were consistent with the histologic subtype of the original RN specimen. Interestingly, one RPLND specimen demonstrated adenocarcinoma; the corresponding RN had been performed over 30 years earlier, and detailed information about the initial pathology was unavailable. Most specimens consisted of confluent masses, which precluded accurate determination of the number of individual lymph nodes involved.

Alternative management options, such as systemic therapy or SABR, are increasingly used in the modern era but are supported mainly by indirect evidence derived from studies of patients with multiple metastases.^3^, ^4^, ^5^ The introduction of immunotherapy‐based systemic regimens, both as adjuvant treatment after RN and in the metastatic setting, has reshaped the therapeutic landscape; however, their role in patients with resectable, node‐only recurrence remains undefined. This study includes patients treated in the current therapeutic era; half underwent RPLND after progression following SABR or systemic therapy failure. Given the limited sample size, it is not appropriate to draw comparisons between surgical or oncologic outcomes in patients undergoing upfront RPLND and those treated after prior therapies. The potential role of RPLND in delaying the need for systemic therapy remains uncertain and warrants further investigation. RPLND in this setting should not be regarded as a competing approach to other strategies but rather as a complementary option within a multimodal management.

This study has several limitations, including its retrospective design and absence of a control group treated with systemic or SABR‐only approaches. The study is limited by its small sample size; however, the inclusion period of only 18 months is relatively short compared with the broader time spans reported in similar publications. This restricted timeframe was intentionally chosen to ensure a contemporary cohort and a minimum follow‐up of 12 months after RPLND. Additionally, most RN were performed outside our institution, leading to potential variability in surgical technique, including the role of nodal resection during RN.

This case series provides contemporary data to a limited body of evidence, showing that RPLND can be performed both in patients refractory to prior therapies and as a frontline option in selected cases, achieving outcomes comparable to those reported in earlier cohorts. It also raises awareness of this surgical option and promotes multicenter collaboration and shared databases to strengthen the evidence base for managing isolated RPLN recurrence in RCC, either in isolation or multimodal.

Further multicenter studies with larger patient populations and standardized selection criteria are warranted to clarify the oncologic benefit of RPLND relative to emerging nonsurgical approaches. Collaborative efforts integrating surgical, radiotherapeutic, and systemic modalities will be essential to define optimal management strategies for this uncommon but clinically relevant pattern of RCC recurrence.

## AUTHOR CONTRIBUTIONS

All authors contributed to the study conception and design, material preparation, data collection analysis and writing of the manuscript. All authors read and approved the final manuscript.

## DISCLOSURES

None.

## CONFLICT OF INTEREST

The authors declare no conflicts of interest.
